# Supplementary material for: Stroke Prediction with Machine Learning Methods among Older Chinese
Source: Int J Environ Res Public Health. 2020 Mar 12;17(6):1828. doi: 10.3390/ijerph17061828 (PMC7142983; doi:10.3390/ijerph17061828)
Supplement: Supplementary file 1 [file ijerph-17-01828-s001.pdf]

**SUPPLEMENTARY MATERIALS**

# Stroke Prediction with Machine Learning Methods among Older Chinese

**Table S1.** Predictors in this study.

| Categories | Variables |
|---|---|
| Demographic | Sex |
| | Age |
| | Comorbidities (hypertension, diabetes, heart disease) |
| Lifestyle | Drinking |
| | Smoking |
| Clinical | High-sensitivity C-reactive protein (hsCRP) |
| | Blood glucose (GLU) |
| | High-density lipoprotein cholesterol (HDLC) |
| | Low-density lipoprotein cholesterol (LDLC) |
| | Triglyceride (TG) |
| | Urea acid (UA) |
| | Systolic blood pressure (SBP) |
| | Diastolic blood pressure (DBP) |

## Data balancing methods used in this study

❖ **Random over-sampling (ROS)**

ROS is the simplest method of over-sampling. The essence is to randomly copy samples in the minority class, therefore achieving consistency with the samples of majority class[1]. As shown in the figure below, the original data ($S$) including the majority class ($S_{maj}$) and minority class ($S_{\min}$) were quite imbalanced. Then, some of these samples in the minority class were randomly copied, as represented by $S^*_{\min}$. So, the samples in the minority class are $S_{\min} + S^*_{\min}$, and the samples in the majority class remain the same. Eventually, these two classes reach a new balance.

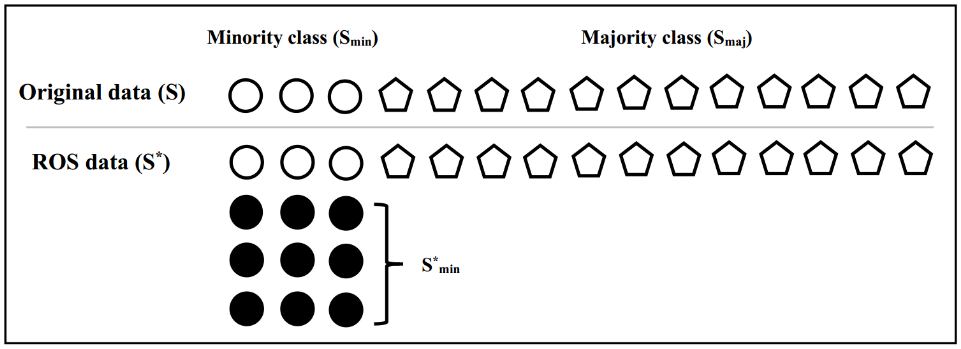


**Supplementary Figure.** The process of random over-sampling.

❖ **Random under-sampling (RUS)**

RUS is the commonly used method of under-sampling. It randomly removes samples from the majority class until the samples are equivalent to each other in the minority and majority classes[1]. As shown in the figure below, the original data ($S$) including the majority class ($S_{maj}$) and minority class ($S_{\min}$) were imbalanced. Then, some of these samples in the majority class were randomly removed. Eventually, these two classes also reach a new balance.

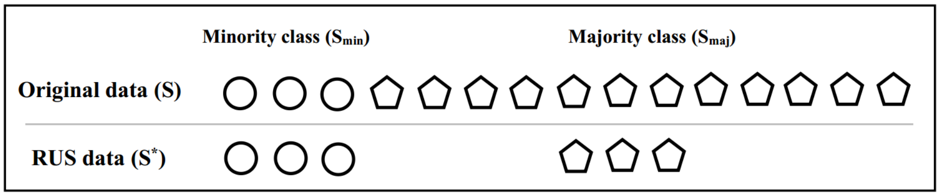


**Supplementary Figure.** The process of random under-sampling.

❖ **Synthetic minority over-sampling technique (SMOTE)**

For synthetic sampling, SMOTE is a powerful tool for dealing with imbalanced data[2]. The SMOTE method creates new artificial samples based on similarities between minority samples[1]. As depicted in the figure below, the circle represents the samples of the minority class ($S_{\min}$). Consider k-nearest neighbors for each sample ($x_i$) in $S_{min}$. Randomly select sample $x_j$ from the k-neighbors. Then, calculate the new samples with the following formula:

$$x_{new} = x_i + D \times \delta$$

Here, $D$ is the distance between $x_i$ and $x_j$, $x_{new}$ represents the new samples created by SMOTE, and $\delta$ represents a random number between [0,1] with the same dimension as $D$.

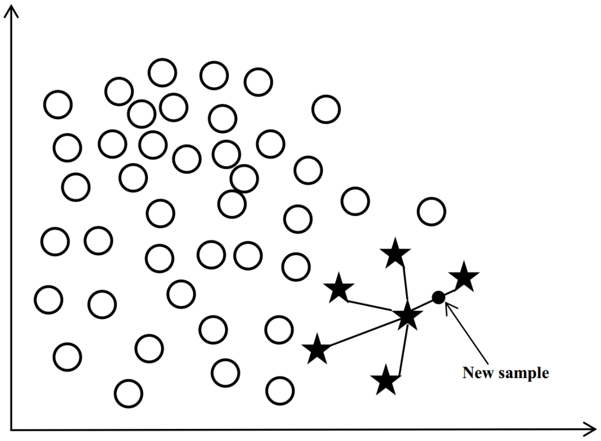


**Supplementary Figure.** The new samples generated by the SMOTE technique.

# fold cross-validation

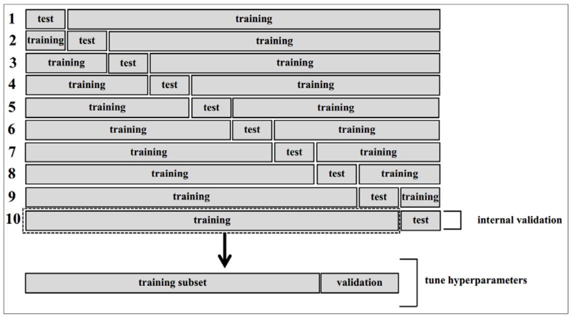


**Figure S1.** The flow chart of 10-fold cross-validation.

**The main process of 10-fold cross-validation**

(1) divide the data set into 10 equal parts without duplication

(2) use the training set for model derivation and parameter tuning

(3) use the testing set for validation of performance

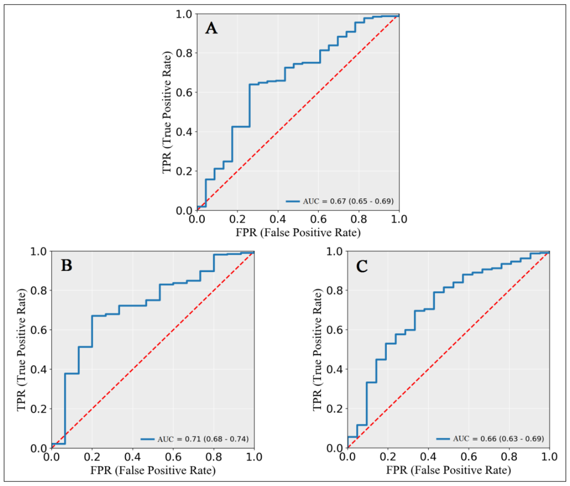


**Figure S2.** ROC curves of the three machine learning methods with the ROS-balanced data set. (**A**) Logistic; (**B**) SVM; (**C**) RF.

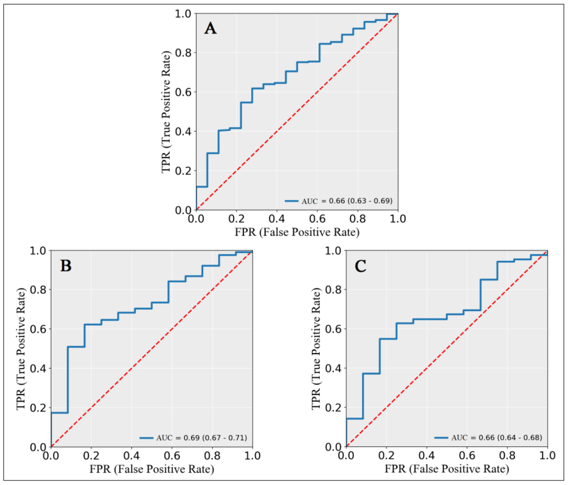


**Figure S3.** ROC curves of the three machine learning methods with the RUS-balanced data set. (**A**) Logistic; (**B**) SVM; (**C**) RF.

## References

1. He H, Garcia EA. Learning from Imbalanced Data. *Ieee T Knowl Data En*. 2009;21:1263-1284
2. Chawla NV, Bowyer KW, Hall LO, Kegelmeyer WP. SMOTE: Synthetic minority over-sampling technique. *J Artif Intell Res*. 2002;16:321-357
